# Current options and future directions of systemic therapy for advanced biliary tract cancer

**DOI:** 10.37349/etat.2021.00054

**Published:** 2021-10-31

**Authors:** Maria Giuseppina Prete, Antonella Cammarota, Antonio D’Alessio, Valentina Zanuso, Lorenza Rimassa

**Affiliations:** 1 Department of Biomedical Sciences, Humanitas University, Via Rita Levi Montalcini 4, 20090 Pieve Emanuele, Milan, Italy; 2 Medical Oncology and Hematology Unit, Humanitas Cancer Center, IRCCS Humanitas Research Hospital, Via Manzoni 56, 20089 Rozzano, Milan, Italy; University of Southampton Faculty of Medicine, UK

**Keywords:** Biliary tract cancer, cholangiocarcinoma, chemotherapy, molecular profiling, driver mutations, targeted therapy, immunotherapy

## Abstract

Biliary tract cancers (BTCs) are aggressive tumors arising from different portions of the biliary tree and classified according to the anatomical location in intrahepatic (i) cholangiocarcinoma (CCA, iCCA), perihilar CCA (pCCA), and distal CCA (dCCA), gallbladder cancer (GBC), and ampulla of Vater cancer (AVC). Due to their silent behavior, BTCs are frequently diagnosed at advanced stages when the prognosis is poor. The available chemotherapeutic options are palliative and unfortunately, most patients will die from their disease between 6 and 18 months from diagnosis. However, over the last decade, amounting interest has been posed on the genomic landscape of BTCs and deep-sequencing studies have identified different potentially actionable driver mutations. Hence, the promising results of the early phase clinical studies with targeted agents against isocitrate dehydrogenase (*IDH*) *1* mutation or fibroblast growth factor (*FGF*) receptor (*FGFR*) *2* aberrations inintrahepatic tumors, and other agents against humanepidermal growth factor receptor (*HER*) *2* overexpression/mutations, neurotrophic tyrosine receptor kinase (*NTRK*) fusions or B-type Raf kinase (*BRAF*) mutations across different subtypes of BTCs, have paved the way for a “precision medicine” strategy for BTCs. Moreover, despite the modest results when used as monotherapy, beyond microsatellite instability-high (MSI-H) tumors, immune checkpoint inhibitors are being evaluated in combination with platinum-based chemotherapy, possibly further expanding the therapeutic landscape of advanced BTCs. This review aims to provide an overview of the approved systemic therapies, the promising results, and the ongoing studies to explore the current and future directions of advanced BTC systemic treatment.

## Introduction

Biliary tract cancers (BTCs) represent a heterogeneous group of rare and aggressive cancers arising from the bile ducts. BTCs include cholangiocarcinoma (CCA), gallbladder cancer (GBC), and ampulla of Vater cancer (AVC). Based on anatomical location, CCA can be classified as intrahepatic CCA (iCCA) and extrahepatic CCA (eCCA), originating from the biliary tree within the liver and outside the liver parenchyma, respectively. Additionally, eCCA is subdivided into perihilar CCA (pCCA) and distal CCA (dCCA) [1, 2].

BTCs account for approximately 3% of all gastrointestinal malignancies [3]. In most countries, BTCs are considered rare with incidence rates below 6/100,000 per year. However, a rising trend over the past four decades has been observed in most countries, mainly due to an increased diagnosis of iCCA [4, 5]. Main risk factors include obesity, cirrhosis, diabetes, viral hepatitis B and C, primary sclerosing cholangitis, gallstones, and, especially in Asian countries, liver flukes [6].

Due to the asymptomatic clinical behavior of the disease, around 60–70% of patients are diagnosed at advanced stages with unresectable or metastatic disease when systemic therapies are the only potential therapeutic options, therefore prognosis remains poor with a 5-year overall survival (OS) of around 5–15% [7, 8].

In recent years, the extensive use of sequencing techniques has revealed a great genomic heterogeneity in the landscape of BTCs. Around half of BTCs are potentially eligible for targeted therapies, thus suggesting the usefulness of incorporating genomic profiling into routine clinical practice [9, 10]. The most relevant mutations with therapeutical implications are isocitrate dehydrogenase (*IDH*) *1* mutation and fibroblast growth factor (*FGF*) receptor (*FGFR*) *2* gene fusions or rearrangements, which are found in up to 15–20% of iCCA [9–11]. They are both more common in women, are mutually exclusive, and are almost never found in extrahepatic BTCs.

Approximately 5% of iCCAs harbor activating serine threonine-protein kinase B-type Raf kinase (*BRAF*) at the *V600E* locus mutations, with the promising activity of dual *BRAF* plus mitogen-activated protein kinase (MAPK)/extracellular signal-regulated kinase (ERK) kinase (MEK) inhibition arising from a phase II multicenter basket trial [12]. The human epidermal growth factor receptor (*HER*) *2* activation can be found in subsets of patients with BTCs. In particular, *HER2* overexpression or gene amplification can occur in up to 15–20% of cases of GBC and eCCA, while rates are low in iCCA [13]. Conversely, *HER2* mutations are much less frequent (1–2% in BTCs), while reaching a 7% rate in AVC [14]. Moreover, fusions in neurotrophic tyrosine receptor kinase (*NTRK*) *1–3* genes are occasionally implicated in BTCs, even though potentially targetable [15]. Similarly, BTCs with microsatellite instability-high (MSI-H)/mismatch repair deficiency (dMMR) account for 1% of the total, representing an infrequent but actionable subgroup [16]. The most relevant targetable aberrations in BTCs are summarized in Table 1.

**Table 1.** Relevant targetable aberrations in BTCs

**Molecular alteration**	**Incidence (%)**	**Anatomical location**	**Investigated agents**
*FGFR2* gene fusions	14–23	iCCA	Pemigatinib Infigratinib Futibatinib Derazantinib Debio 1347 Erdafitinib
*IDH* mutations	7–20 (*IDH1*) 3 (*IDH2*)	iCCA	Ivosidenib Enasidenib Dasatinib FT-2102
*HER2* overexpression/amplification	15	GBC, eCCA	Trastuzumab Trastuzumab-pertuzumab Trastuzumab deruxtecan T-DM1 Zanidatamab
*HER2* mutations	1–2	GBC, eCCA, iCCA	Neratinib
	7	AVC	
BRAF V600E mutation	< 5	iCCA	Dabrafenib-trametinib
*NTRK* fusions	3.5	iCCA	Larotrectinib Entrectinib
MSI-H	1	eCCA, iCCA, GBC	Pembrolizumab

T-DM1: trastuzumab-emtansine; FT-2102: olutasidenib

Other relevant alterations are the activation of the Janus kinase/signal transducer and activator of transcription (JAK/STAT) signaling pathway, which can occur in 58–77% of iCCAs, and gain of function mutations in protein tyrosine phosphatase non-receptor type 3 (PTPN3) [17]. Moreover, mutations in DNA-damage repair (*DDR*) genes are found in about 20% of BTCs, especially in extrahepatic BTCs [8]. Lastly, although observed in a very small percentage of BTCs (< 5%) wingless/integrated (WNT) pathway alterations such as ring finger protein 4 (RNF4) mutations are currently under investigation [13]. Therefore, the identification of molecular alterations with consequent personalized treatment options should become routine standard practice in BTCs with the ultimate goal of improving survival.

In light of this evolving scenario, the aim of this review is to provide an overview of the current options and future directions of systemic treatment for advanced BTCs.

## Systemic treatment for BTCs

### Conventional chemotherapy

#### First-line

Cytotoxic chemotherapy represents the backbone of treatment for unresectable and metastatic BTCs. Up to the last decade, no definitive recommendations on first-line regimen were available due to a lack of robust evidence. In 2010, the ABC-02 study set cisplatin and gemcitabine regimen as the standard of care for advanced BTCs. This phase III trial showed a statistically significant improvement in OS with a 3-month survival advantage [hazard ratio (HR) = 0.64, 95% confidence interval (CI): 0.52–0.80; *P* < 0.001] in all subsites, over single-agent gemcitabine [18]. These data were confirmed by the similar Japanese BT-22 study [19], and by a meta-analysis of the aforementioned trials [20]. More recently, a randomized phase III trial conducted in Japan (FUGA-BT trial) demonstrated the non-inferiority of gemcitabine plus S-1 (Tegafur/gimeracil/oteracil) compared to the standard of care in the first-line setting [21]. In addition, the KHBO1401-MITSUBA study directly compared gemcitabine, cisplatin, and S-1 to gemcitabine and cisplatin, demonstrating modest survival benefit [22].

Several clinical trials are currently ongoing to determine whether the intensification of chemotherapy could be an appropriate strategy. A phase II study evaluated the efficacy of the triplet gemcitabine, cisplatin, and nab-paclitaxel showing median progression-free survival (PFS) and median OS of 11.8 (95% CI: 6.0–15.6) and 19.2 [95% CI: 13.2-not estimable (NE)] months, respectively. Only 16% of patients withdrew owing to adverse events (AEs), despite the high rate (58%) of grade ≥ 3 AEs [23]. This regimen is being evaluated against cisplatin and gemcitabine in the ongoing Phase III SWOG 1815 trial (NCT03768414). The use of 5-fluorouracil (5-FU)/leucovorin/irinotecan/oxaliplatin (FOLFIRINOX) as first-line treatment has led to a disease control rate (DCR) of 75% and OS of 15 months in small retrospective series [24, 25]. Similarly, the phase II/III PRODIGE38-AMEBICA trial compared modified FOLFIRINOX *vs.* cisplatin and gemcitabine, without reaching its primary endpoint of PFS rate at 6-months [26]. The NIFE phase II trial aims to challenge the current first-line therapy using nanoliposomal irinotecan (nal-IRI)/5-FU/leucovorin (NCT03044587). Lastly, the phase Ib ABC-08 trial evaluated cisplatin in combination with acelarin (NUC-1031), a phosphoramidate variant of gemcitabine that bypasses the basic resistance mechanism to the nucleoside analog [27]. Based on a remarkable objective response rate (ORR) of 63.6%, the combination is under investigation in a phase III trial (NCT04163900) as first-line treatment.

#### Second line

Until recently, the efficacy of second-line therapy in patients with BTCs was unproven. The phase III ABC-06 study assessed the advantage of modified oxaliplatin/5-FU/leucovorin (mFOLFOX) over active symptom control (ASC) in patients failing cisplatin and gemcitabine. Although the median OS improvement was modest, 6.2 months (95% CI: 5.4–7.6) in the ASC plus mFOLFOX group *vs.* 5.3 months (95% CI: 4.1–5.8) in the ASC alone group (HR = 0.69; 95% CI: 0.50–0.97; *P* = 0.031), a clinically meaningful 15% survival improvement was observed at 12-month [25.9% (95% CI: 17.0–35.8) *vs.* 11.4% (95% CI: 5.6–19.5)] [28]. Therefore, mFOLFOX is currently being considered the second-line standard-of-care chemotherapy. In a Dutch single-arm phase II study, promising results arose from the use of FOLFIRINOX in selected patients after cisplatin and gemcitabine, with a median OS of more than 18 months [29]. Similarly, the AIO NALIRICC study is currently investigating the efficacy of nal-IRI in combination with 5-FU *vs.* 5-FU alone in the second-line setting (NTC03043547). Unfortunately, an escalation to triplet chemotherapy regimens is highly unlikely in clinical practice due to the rapid worsening of patient performance status.

### Targeted therapy

Deep-sequencing studies have shed some light on the highly complex molecular biology driving BTCs, identifying potentially targetable genetic alterations [30, 31]. In a large comprehensive study, nearly 40% of BTCs patients were found to harbor actionable genetic alterations and interestingly the genetic driver mutations vary between the different subtypes of CCA [30, 32]. Furthermore, in the BTC subgroup [*n* = 43/1,035 (4%)] of the MOSCATO-01 trial encouraging longer survivals (median OS 17 *vs.* 5 months; HR = 0.29; 95% CI: 0.11–0.76; *P* = 0.008) were achieved by patients who received targeted therapy matched to the somatic alteration found in their tumors compared with those treated with unselected therapies, paving the way for further investigations of a personalized targeted approach, especially for iCCA [33].

#### FGF pathway inhibitors

One of the main targetable genetic alterations identified in iCCA is that of *FGFR* [30, 31, 33, 34]. Of the 5 known isoforms of FGFRs (FGFR1-5), FGFR5, lacking in the tyrosine kinase domain, is not considered relevant in carcinogenesis [35]. Among FGFR aberrations, *FGFR2* gene fusions or rearrangements are the most common type, occurring in 14–23% of iCCA, with bicaudal C homolog 1 (BICC1) protein being the most frequent FGFR2 rearrangement partner (29.7%) [34, 36]. Furthermore, confirming findings from prior studies of whole-genome and targeted exon sequencing of iCCA, *FGFR2* gene fusion/rearrangement has been reported as mutually exclusive with IDH1 mutations, another key actionable alteration found in iCCA [36, 37].

##### Pemigatinib

Showing promising antitumor activity in preclinical studies, pemigatinib, a selective oral inhibitor of FGFR1-3, has been further investigated in the FIGHT-202 trial, an open-label, multicohort, single-arm, phase II study [38]. The study enrolled 146 patients with previously treated, metastatic, or unresectable locally advanced CCA, with or without FGFR aberrations within three cohorts (cohort 1: 107 patients with *FGFR2* gene fusions or rearrangements; cohort 2: 20 patients with other FGF/FGFR alterations; cohort 3: 18 patients with no FGF/FGFR alterations). Enrolled patients received a starting dose of 13.5 mg oral pemigatinib once daily on a 21-day cycle (2 weeks on, 1 week off) until disease progression or unacceptable toxicity. The primary endpoint was centrally assessed ORR per response evaluation criteria in solid tumors (RECIST) v1.1 [39] in patients with *FGFR2* gene fusions or rearrangements. After a median follow-up of 17.8 months, the study met its primary endpoint with a reported ORR of 35.5% (95% CI: 26.5–45.4), with 3 complete response (CR), and a DCR of 82% in carriers of *FGFR2* fusions or rearrangements. The median duration of response (DOR) was 7.5 months (95% CI: 5.7–14.5). No patients with other FGF/FGFR alterations or without FGF/FGFR alterations achieved a response. The most common AE was hyperphosphatemia (60%), an expected effect of FGFR inhibition. The most frequent grade ≥ 3 AEs were hypophosphatemia (12%), arthralgia (6%), and stomatitis (5%). Additionally, serous retinal detachment occurred in 4% of the patients, mandating cautious ophthalmological monitoring during treatment [38]. Given the promising results of FIGHT-202, the phase III FIGHT-302 study investigating the efficacy of pemigatinib *vs.* standard chemotherapy in the first-line setting in patients with *FGFR2* gene fusions or rearrangements is ongoing (Table 2) [40]. Of note, pemigatinib has been approved in May 2020 by the United States Food and Drug Administration (FDA) and in March 2021 by the European Medicines Agency (EMA) for patients with metastatic or locally advanced unresectable CCA harboring *FGFR2* gene fusions/rearrangements failing at least one line of systemic therapy.

**Table 2.** Ongoing first-line phase III trials with targeted agents for advanced BTCs

**Study name/number**	**Target population**	**Estimated sample size**	**Experimental treatment**	**Comparator**	**Primary endpoint**	**Secondary endpoints**
FIGHT-302^1^ NCT03656536	*FGFR2* rearrangements	432 patients	Pemigatinib 13.5 mg QD on a 3-week cycle	Cisplatin 25 mg/mq + gemcitabine 1000 mg/mq on days 1 and 8 q3w up to 8 cycles	PFS per RECIST v1.1 by ICR	OS, ORR, DOR, DCR per RECIST v1.1 by ICR, safety, QoL
PROOF trial NCT03773302	*FGFR2* fusions	384 patients	Infigratinib 125 mg orally QD, 3 weeks on, 1 week off	Cisplatin 25 mg/mq + gemcitabine 1000 mg/mq on days 1 and 8 q3w up to 8 cycles	PFS per RECIST v1.1 by ICR	OS, ORR, DOR, BOR, DCR per RECIST v1.1 by ICR, PFS per investigator assessment, safety
FOENIX-CCA3^1^ NCT04093362	*FGFR2* rearrangements	216 patients	Futibatinib 20 mg orally QD on a 3-week cycle	Cisplatin 25 mg/mq + gemcitabine 1000 mg/mq on days 1 and 8 q3w up to 8 cycles	PFS per RECIST v1.1 by ICR	OS, ORR, DCR per RECIST v1.1 by ICR, PFS per investigator assessment, safety

1 Crossover is allowed in FIGHT-302 and FOENIX-CCA3; QD: once a day; q3w: every 3 weeks; ICR: independent central review; QoL: quality of life; BOR: best overall response

##### Infigratinib

In a single-arm, phase II study infigratinib, an oral pan-FGFR selective inhibitor given orally at a dose of 125 mg daily for 21 days of 28-day cycles, was proven effective in previously treated patients with metastatic or unresectable CCA and FGFR aberrations, reaching a centrally reviewed ORR of 23.1% (95% CI: 15.6–32.2), including 1 CR, in patients with *FGFR2* fusions/rearrangements, which was the primary endpoint of the study [41, 42]. Interestingly, in a prespecified subgroup analysis centrally reviewed ORR was 34% in the second-line setting and < 16% in the third- or later-line setting. The toxicity profile of infigratinib was consistent with the findings reported for this drug class, with the most common AEs being hyperphosphatemia (77%), despite all patients received prophylaxis with the oral phosphate binder sevelamer [42]. Based on these results infigratinib has been approved in May 2021 by the FDA for patients with previously treated, unresectable locally advanced, or metastatic CCA with *FGFR2* fusion or other rearrangements as detected by an FDA-approved test. Due to these encouraging results, the phase III PROOF trial evaluating the first-line infigratinib *vs.* standard chemotherapy in patients with inoperable CCA harboring *FGFR2* fusions or rearrangements is underway (Table 2) [43].

##### Futibatinib

Futibatinib is an oral FGFR1-4 highly selective irreversible inhibitor administered orally at a dose of 20 mg once daily in 21-day cycles [44]. A recently presented interim analysis of the FOENIX-CCA2 phase II study reported an ORR of 37.3% (including 1 CR), the primary endpoint of the study, among 103 previously treated patients with metastatic or advanced unresectable iCCA carrying *FGFR2* fusions (82.1%) or other rearrangements [45, 46]. The most common treatment-related AEs (TRAEs) were hyperphosphatemia (79.1%), diarrhea (37.3%), and dry mouth (32.8%). Interestingly, higher phosphate levels showed a trend in responders *vs.* non-responders [46]. Of note, futibatinib has also shown activity in patients with FGFR aberrations (ORR 17.6%) other than *FGFR2* fusions, and even in progressors on previous FGFR inhibitors (ORR 30.8%), suggesting that it may overcome the mechanisms of resistance [47]. These results prompted the initiation of the multicenter, open-label, randomized phase III FOENIX-CCA3 trial, investigating the first-line futibatinib *vs.* standard chemotherapy in patients with metastatic or unresectable iCCA harboring *FGFR2* rearrangements (Table 2).

##### Derazantinib

Derazantinib, an oral FGFR1-3 inhibitor, was tested in a small phase I/II study enrolling patients with *FGFR2* gene fusions positive metastatic or unresectable iCCA. Administered orally at a dose of 400 mg and 300 mg once daily continuously in phase I and in the phase II part, respectively, derazantinib provided an ORR of 21% with a DCR of 83% in patients with *FGFR2* gene fusions [48]. Since a post-hoc analysis of this study reported a DCR of 67% in 6 patients carrying *FGFR2* amplification or mutations, derazantinib efficacy was evaluated in other settings besides *FGFR2* fusions [49]. Therefore, an open-label, single-arm, phase II study (FIDES-01) [50] is enrolling previously treated patients with unresectable iCCA within two cohorts according to the FGFR2 aberration harbored (cohort 1: *FGFR2* gene fusions carriers; cohort 2: *FGFR2* mutations and amplifications carriers). In addition to the promising preliminary results (ORR 24%) supporting the clinical value of derazantinib in the *FGFR2* gene fusions cohort [51], an encouraging DCR of 79% with 1 confirmed CR has just been reported in an interim analysis on 14 patients from the cohort with FGFR2 mutations and amplification [52]. Concordantly, a pooled interim analysis in a sizeable proportion of patients (*n* = 23) with iCCA harboring FGFR2 mutations or amplifications treated with derazantinib in the studies ARQ 087-101 (NCT01752920), FIDES-01 (NCT03230318) and in the early access program (EAP; NCT04087876) and compassionate use program has provided a median PFS of 7.2 months (95% CI 4.6–11.1) with a median DOR of 8.2 months (95% CI 4.9–11.1). The toxicity profile was in line with that of the other agents from the same drug class, with hyperphosphatemia being the most common AE. Interestingly, derazantinib treatment was associated with a low incidence of grade ≥ 3 nail toxicity, retinopathy, hand-foot syndrome and stomatitis [53].

##### Debio 1347

Debio 1347 is another selective oral inhibitor of FGFR1-3. In a small subset of CCA patients (*n* = 5) with FGFR aberrations treated with Debio 1347 at the dose of 80 mg orally once daily continuously in 28-day cycles in the expansion phase of a first-in-human, open-label study, only patients with *FGFR2* gene fusions showed a benefit, with 2 stable diseases (SD) and 2 partial responses (PR) [54]. Thus, Debio 1347 is being further evaluated in previously treated *FGFR* fusion-positive advanced solid tumors in the open-label, multicenter, phase 2 FUZE trial [55].

##### Erdafitinib

Erdafitinib, an oral potent inhibitor of all four FGFR family members and other highly related kinases, is being evaluated in different solid tumors with FGFR2 aberrations in an ongoing, open-label, phase IIa study [56, 57]. Administered at a dose of 8 mg orally once daily continuously in 28-day cycles, up-titrated to 9 mg in patients not experiencing hyperphosphatemia during cycle 1, erdafitinib provided a remarkable ORR of 47% (all PRs) and a DCR of 80% in the cohort of Asian patients (*n* = 15) with previously treated advanced CCA and FGFR alterations (mostly *FGFR* fusions), warranting further evaluation in this setting [57].

While more drugs targeting this pathway are under development, amounting interest is growing on the possible prognostic and predictive role of FGFR aberrations. In this context, two retrospective analyses evaluated how the FGFR2 alterations eventually influenced the response to prior standard chemotherapy, reporting similar PFS on both first-line and second-line chemotherapy among the *FGFR2* fusion/rearrangement carriers compared with the FGFR2 wild-type counterparts [58, 59]. Despite these findings, a longer median OS was reported in patients with FGFR2 aberrations (31.3 months; 95% CI: 5.8-NE) compared with FGFR2 wild-type patients (21.8 months; 95% CI: 16.7–26.6), supporting the positive prognostic role of FGFR alterations [59].

#### IDH inhibitors

Several tumor types acknowledge *IDH* mutations as pathogenetic alterations, most notably gliomas and acute myeloid leukemia [60]. *IDH1* mutations are found in 7–20% of iCCA, while *IDH2* mutations in 3% [15, 30, 61].

Currently, several trials are testing different IDH inhibitors for the treatment of iCCA: inhibitors of IDH1, IDH2, and pan-IDH1/2. The first promising results came from early phase clinical trials; in particular, an IDH1 inhibitor, ivosidenib, given at a 500 mg daily dose, was first successfully tested in a phase I study on 77 patients with previously treated CCA harboring *IDH1* mutations [62]. Thereafter, ivosidenib was tested against placebo as second- or third-line treatment in phase III ClarlDHy trial, enrolling 185 patients with metastatic *IDH1*-mutated CCA, with PFS as primary endpoint [63]. Median PFS in the ivosidenib arm was statistically significantly longer compared to the placebo arm (2.7 *vs.* 1.4 months; HR = 0.37; 95% CI: 0.25–0.54; *P* < 0.0001). The mature data for OS showed a trend in favor of ivosidenib despite the high rate (70%) of crossover: median OS was 10.3 months and 7.5 months in the ivosidenib and placebo arm, respectively (HR= 0.79; 95% CI: 0.56–1.12; *P* = 0.093) [64]. Thecrossover-adjusted median OS for placebo was 5.1 months (HR = 0.49; 95% CI: 0.34–0.70; *P* < 0.0001), showing the OS advantage of ivosidenib.

The most common all-grade AEs were nausea (41%), diarrhea (35%), and fatigue (31%), with 7% of patients in the ivosidenib arm and 9% in the placebo arm experiencing a grade ≥ 3 AE and 7% and 0% discontinuing treatment due to an AE, in the ivosidenib and in the placebo arm, respectively [64]. Of note, ivosidenib has been approved in August 2021 by the FDA for patients with previously treated, locally advanced, or metastatic CCA with an *IDH1* mutation as detected by an FDA-approved test. Furthermore, an ongoing phase II study is testing the combination of ivosidenib and nivolumab in *IDH1*-mutant advanced solid tumors (NCT04056910). Concerning the inhibition of IDH2, several drugs are currently under investigation in early phase trials including iCCA, namely enasidenib (NCT02273739), a pure IDH2 inhibitor, and dasatinib (NCT02428855), AG-881 (NCT02481154), and FT-2102 (NCT03684811), which are IDH1/2 inhibitors.

#### BRAF/MEK inhibitors

BRAF and MEK are two key oncogenic proteins of the MAPK signal transduction cascade and their activating mutations are found in a wide range of cancers, such as melanoma and colorectal cancer, and specific therapies target the most common *BRAF* mutation, V600E [65]. The mutation is found in less than 5% of CCA, especially in iCCA [15].

In an unselected population, the association of binimetinib, a MEK inhibitor, and chemotherapy did not achieve an improvement in PFS in a phase I/II trial [66], nor did the association of another MEK inhibitor, selumetinib, and chemotherapy in a phase II trial [67]. After the first pioneering results of vemurafenib, a BRAF inhibitor, used as a single agent in different *BRAF*-mutant cancers [68, 69], the most recent results came from the combined blockade of BRAF and MEK with daBRAFenib and trametinib, respectively, in the phase II ROAR trial. In this basket trial, patients with different tumor types harboring V600E *BRAF* mutation received the two drugs, and in the BTC cohort the experimental treatment obtained an impressive ORR of 47% (95% CI: 31–62) in heavily pretreated patients, with a median PFS of 9 months (95% CI: 5–10), and a median OS of 14 months (95% CI: 10–33), suggesting a decisive predictive role of *BRAF* mutation in BTCs [12].

#### HER2 overexpression/amplification and mutations

HER2 overexpression/amplification is found in 10–15% of BTCs, especially in GBC [30]. In previously treated, chemotherapy-refractory patients harboring *HER2* amplification, previous evidence supported the approach of a double blockade with the combination of pertuzumab and trastuzumab [70]. Recently, MyPathway, a non-randomized, multicenter, open-label, phase 2a basket trial, assessed the efficacy of this dual anti-HER2 regimen in 39 metastatic BTC patients with HER2 amplification, overexpression, or both. This study showed an interesting ORR (23%, 95% CI: 11–39) with 9 patients achieving PR, and a DCR of 51% (95% CI: 35–68) with 11 patients with SD longer than 4 months (range 4.2–22.7 months). Median DOR was 10.8 months (95% CI: 0.7–25.4). The combination was well tolerated with no serious TRAEs, and no treatment discontinuations or deaths due to AEs. TRAEs occurred in 62% of the patients and the most common were diarrhea (26%), increased alanine aminotransferase, increased aspartate aminotransferase, and infusion-related reaction (10% each). Grade 3 TRAEs, including biochemical liver alterations, were reported in three patients [71].

The combination of chemotherapy plus trastuzumab is currently under study in a larger clinical trial (NCT03613168). Furthermore, another promising anti-HER2 drug is trastuzumab deruxtecan (DS-8201), an antibody-drug conjugate enriched by a topoisomerase I inhibitor, which is now under investigation in phase II clinical study [72]. Other anti-HER2 agents currently under investigation as monotherapy for *HER2*-amplified BTCs are trastuzumab (NCT00478140), T-DM1 (NCT02999672), and zanidatamab (NCT04466891). Of note, neratinib is currently being tested for *HER2*-mutated BTCs in the phase II SUMMIT trial, where it achieved an ORR of 12% (95% CI: 3–31) in 25 evaluable patients [14].

#### Regorafenib

Regorafenib is an oral multi-kinase inhibitor (MKI) targeting the vascular endothelial growth factor (VEGF) receptor (VEGFR), the platelet-derived growth factor receptor (PDGFR)-β, and FGFR1, which play a key role in tumor angiogenesis and metastasis. This drug was tested in phase II studies for chemotherapy-refractory BTC patients, showing promising ORR and DCR [73, 74]. Recently, regorafenib demonstrated an advantage over placebo in terms of PFS in the REACHIN randomized phase II study [75], with a median PFS of 3 months (95% CI: 2.3–4.9) in the interventional group *vs.* 1.5 months (95% CI: 1.2–2.0) in the placebo group (HR = 0.49, 95% CI: 0.29–0.81; *P* = 0.004), while median OS did not significantly differ [5.3 mocnths (95% CI: 2.7–10.5) *vs.* 5.1 months (95% CI: 3.0–6.4), *P* = 0.28]. The combination of regorafenib plus gemcitabine and oxaliplatin has been tested in the phase II BREGO trial, whose results are awaited [76]. The identification of predictive biomarkers of response could allow a better patients selection, and some evidence showed a possible role of mucosa-associated lymphoid tissue lymphoma translocation protein 1 (MALT-1) expression in iCCA both as a target and as a prognostic factor for regorafenib treatment [77]. Also, increased levels of VEGF-D, interleukin-6, and glycoprotein 130 were linked to a worse survival during regorafenib therapy [78].

#### NTRK inhibitors

*NTRK* gene encodes for tropomyosin receptor kinase (TRK), which is linked to the MAPK pathway when activated. *NTRK* activating fusions are found in around 3.5% of iCCA [78] and can constitute a therapeutic target for NTRK inhibitors (i.e., entrectinib or larotrectinib). These drugs have shown outstanding results in terms of ORR [79, 80], leading to their agnostic site approval by the FDA in all tumor types harboring *NTRK* gene fusions, regardless of histology. Currently, clinical trials are evaluating these drugs in large populations, including advanced BTCs (NCT02576431, NCT02568267).

### Immunotherapy

Besides targeted therapy, also immune checkpoints inhibitors (ICI) have been tested in advanced BTCs. However, findings on the clinical use of immunotherapy as a single-agent showed only modest efficacy in unselected patients. For this reason, several combinations of systemic therapies with ICI are now being tested and specific biomarkers are being explored. Indeed, a crucial challenge is represented by the lack of validated predictive biomarkers that could help to identify responders to immunotherapy. Due to the heterogeneity and complexity of BTCs, the prevalence of the well-known biomarkers such as programmed cell death 1 (PD-1)/programmed cell death ligand 1 (PD-L1) expression, tumor mutational burden (TMB), and MSI/dMMR is still unclear. Moreover, except for MSI/dMMR, their predictive role is yet to be established [81].

#### Pembrolizumab

Pembrolizumab, an anti-PD-1 monoclonal antibody (mAb) has been the first ICI tested in advanced BTCs. The KEYNOTE-028 was a multi-cohort phase Ib trial enrolling previously treated patients with PD-L1-positive advanced solid tumors, including 24 BTC patients (20 with CCA and 4 with GBC). In the BTC cohort, the ORR, which was the primary endpoint of the study, was 13 % with 3 PR, while median PFS and OS were 1.8 months (95% CI: 1.4–3.1) and 5.7 months (95% CI: 3.1–9.8), respectively [82]. The following KEYNOTE-158 was a basket trial including the highest number of BTC patients treated with immunotherapy after the first-line failure so far. In this study, pembrolizumab revealed disappointing results in 104 BTC patients with a low ORR of 5.8%, a median PFS of 2 months, and a median OS of 9.1 months without a clear correlation with PD-L1-combined positive score (CPS) [83]. However, Marabelle et al. [84] reported the results achieved in patients with previously treated MSI-H/dMMR non-colorectal cancer enrolled in the trial, including 22 patients with BTCs (all CCA). In the BTCs cohort pembrolizumab resulted in an ORR of 40.9% with 2 CRs, a median PFS of 4.2 months, and a median OS of 24.3 months. Therefore, despite being rare in BTCs (approximately 1%) [85–87], the MSI-H status represents the only reliable predictive biomarker of clinical response to immunotherapy so far.

#### Nivolumab

Nivolumab, another anti-PD-1 mAb, also showed modest efficacy as monotherapy in patients with refractory advanced BTCs. In a recently published phase II study testing nivolumab in 54 refractory microsatellite-stable BTC patients, ORR, the primary endpoint of the study, was 22% with a DCR of 59%. Median OS and PFS were 14.2 months [95% CI: 6.0-not reached (NR)] and 3.7 months (95% CI: 2.3–5.7), respectively [88].

Similar results were shown in one of the two cohorts of a Japanese phase I study assessing efficacy and tolerability of nivolumab, as monotherapy in patients refractory or intolerant to gemcitabine-based treatment regimens, or in combination with cisplatin and gemcitabine in chemotherapy-naive patients. Nivolumab as a single-agent resulted in very limited results in terms of median OS (5.2 months; 90% CI: 4.5–8.7) and PFS (1.4 months; 90% CI: 1.4–1.4) with only one patient (with Lynch syndrome, thus MSI-H) having an objective response [89].

#### Durvalumab

Durvalumab is a PD-L1 inhibitor that has shown limited efficacy in refractory advanced BTCs when tested as monotherapy or in combination with tremelimumab, an anti-cytotoxic T-lymphocyte-associated antigen 4 (CTLA-4) mAb, in early phase studies [90, 91]. However, in a recent phase, II study assessing durvalumab with or without tremelimumab in addition to first-line standard chemotherapy in Asian BTC patients, more promising results were achieved [92]. In detail, ORR, the primary endpoint of the study, was 73.4% and 73.3% with remarkable DCR of 98% and 100%, in durvalumab plus chemotherapy and durvalumab/tremelimumab plus chemotherapy, respectively. Furthermore, a median PFS of 11.9 months (95% CI: 10.1–13.7) and a median OS of 20.7 months (95% CI: 13.8–27.6) were reached in the combination of dual ICI plus chemotherapy. Of note, in this study the baseline tissue TMB did not correlate with PFS or OS. The efficacy of this combination strategy will need to be confirmed also in Caucasian BTC patients.

#### Immunotherapy combinations

Considering the modest results with ICI as monotherapy in advanced BTCs, a mounting interest has been posed on the combination of ICI with other agents. ICIs have been combined with antiangiogenic agents with controversial results. In a phase I study the combination of pembrolizumab plus the anti-VEGFR2 ramucirumab achieved a median OS of 6.4 months (95% CI: 4.2–13.3) and a median PFS of 1.6 (95% CI: 1.4–2.8) months in the second-linesetting [93]. Conversely, the results of the BTCs cohort (*n* = 31) of the phase II LEAP-005 trial, testing the combinationof pembrolizumab plus the MKI lenvatinib, appeared more promising, with a DCR of 68% (95% CI: 49–83), a median OS of 8.6 months (95% CI: 5.6-NR), and a median PFS of 6.1 months (95% CI: 2.1–6.4) in patients who had received one prior line of therapy [94]. Among phase I-II studies assessing immunotherapy in combination with chemotherapy, camrelizumab (SHR-1210), an anti-PD-1 antibody, has been assessed as first-line treatment in association with gemcitabine and oxaliplatin in a single-arm phase II study. Among 37 Asian patients, 20 patients had a PR (54%), 13 SD (35%), and 3 progressive diseases (PD, 8%) at best. The median PFS and OS were 6.1 months and 11.8 months, respectively [95]. Moreover, in the aforementioned Japanese phase I study nivolumab showed better efficacy results in first-line when combined with chemotherapy, reaching a median OS of 15.4 months (90% CI: 11.8-NE) and a median PFS of 4.2 months (90% CI: 2.8–5.6) with 11 out of 30 patients having an objective response [89]. Focusing on the currently ongoing phase II-III trials in the first-line setting (Table 3), the phase III TOPAZ-1 (NCT03875235) and KEYNOTE-966 (NCT04003636) are evaluating the addition of durvalumab [1500 mg intravenously (i.v.) q3w, when combined to chemotherapy, thereafter every 4 weeks] or pembrolizumab (200 mg i.v. q3w), respectively, to the standard-of-care gemcitabine plus cisplatin. In addition, the phase II randomized IMbrave151 trial is testing atezolizumab with or without the anti-VEGF mAb bevacizumab, in combination with cisplatin plus gemcitabine (CisGem, NCT04677504). Moreover, based on encouraging efficacy observed in a phase I study, M7824, a bifunctional fusion protein that simultaneously targets the transforming growth factor-β (TGF-β) and the PD-L1, is currently being evaluated in combination with standard chemotherapy in phase II/III randomized trial (NCT04066491).

**Table 3.** Ongoing first-line randomized phase II–III trials with ICI for advanced BTCs

**Study name/number**	**Phase**	**Estimated sample size**	**Experimental treatment**	**Comparator**	**Primary endpoint**	**Secondary endpoints**
IMbrave151 NCT04677504	II	150 patients	Atezolizumab plus bevacizumab plus CisGem^1^	Atezolizumab plus placebo plus CisGem^1^	PFS per RECIST v1.1 by the investigator	OS, ORR, DOR, DCR per RECIST v1.1 by investigator, TTCD, safety, ADAs for atezolizumab
NCT04066491	II–III	512 patient	Bintrafusp alfa (M7824) plus CisGem^1^	Placebo plus CisGem^1^	Safety run-in part: DLTs Double-blinded part: OS	ORR, DOR, PFS per RECIST v1.1 by investigator, safety, Bintrafusp alfa PK, ADAs for Bintrafusp alfa
TOPAZ-1 NCT03875235	III	757 patients	Durvalumab plus CisGem^1^	Placebo plus CisGem^1^	OS	PFS, ORR, DOR per RECIST v1.1 by ICR and by investigator, OS by PD-L1 expression, PK of durvalumab, ADAs for durvalumab, QoL
KEYNOTE-966 NCT04003636	III	1048 patients	Pembrolizumab plus CisGem^1^	Placebo plus CisGem^1^	OS	ORR, DOR, PFS per RECIST v1.1 by ICR, safety
NCT03478488	III	480 patients	KN035 plus GEMOX^2^	GEMOX^2^	OS	PFS, ORR, DCR, DOR, TTP per RECIST v1.1 by ICR

1 CisGem: cisplatin 25 mg/mq + gemcitabine 1000 mg/mq intravenously (i.v.) on day 1 and 8 on a 21-day cycle up to 8 cycles; ^2^GEMOX: gemcitabine 1000 mg/mq on day 1 and 8 and oxaliplatin 85 mg/mq i.v. on day 1 of a 21-day cycle up to 6 cycles. TTCD: time to clinical deterioration; ADAs: anti-drug antibodies; DLTs: dose-limiting toxicities; PK: pharmacokinetics; TTP: time to progression

In the Asian population, a phase III trial is currently randomizing patients to receive gemcitabine plus oxaliplatin (GEMOX) with or without KN035, an anti-PD-L1 mAb (NCT03478488). Hopefully, the eagerly awaited results of the currently ongoing trials testing ICI combinations will potentially improve the prognosis of patients with such rare cancers.

## Conclusions

Despite chemotherapy has represented the mainstay in advanced BTCs over the last years, the molecular characterization of these malignancies, showing potentially actionable mutations, has paved the way for a precision medicine approach. Among all, drugs targeting FGFR2 aberrations and IDH1 mutations have shown to upgrade the management of molecularly selected advanced BTC patients. Indeed, pemigatinib, recently approved in previously treated advanced CCA patients with FGFR2 aberrations, represents the first targeted therapy to be introduced as a standard treatment for these malignancies. Moreover, thanks to the positive findings of the ClarIDHy trial, ivosidenib will be soon a further option in BTC patients with IDH1 mutation failing previous treatment lines, thus contributing to expanding the current armamentarium of systemic treatments. Besides FGFR2 and IDH1 aberrations, the therapeutic significance of other rare but targetable alterations, such as BRAF mutations, HER2 overexpression or mutations, NTRK fusions, or MSI-H/dMMR, warrants the implementation of molecular testing in clinical practice for patients with advanced disease. Nevertheless, the increasing evidence on therapeutic resistance to targeted agents will pose novel challenges. Notably, recent preclinical studies have shown that non-coding RNA (ncRNA) plays a crucial role in the oncogenesis of BTCs revealing the huge potential of an RNA-based therapy. However, research and technological advancements in this field are still at an early stage and clinical validation of preclinical findings is still missing [96].

With the notable exception of immunotherapy for MSI-H BTC patients, ICI monotherapy provided only modest advances and more promising results are awaited from ICI combinations. Unanswered questions still remain regarding which subgroups of patients will derive major benefits from immunotherapy considering the lack of reliable predictive biomarkers among microsatellite stable advanced BTCs.

Finally, while looking for novel therapeutic options, BTC remains a tricky disease with most patients diagnosed at advanced stages and several unmet needs. Only the multidisciplinary expertise in dedicated centers, highly recommended in the management of BTC patients, can strengthen the link between basic and clinical science, and therefore hopefully improve patient care and prognosis.

## Abbreviations

5-FU: 5-fluorouracil
ADAs: anti-drug antibodies
AEs: adverse events
ASC: active symptom control
AVC: ampulla of Vater cancer
BRAF: B-type Raf kinase
BTCs: biliary tract cancers
CCA: cholangiocarcinoma
CI: confidence interval
CisGem: cisplatin plus gemcitabine
CR: complete response
DCR: disease control rate
dMMR: mismatch repair deficiency
DOR: duration of response
eCCA: extrahepatic cholangiocarcinoma
FDA: Food and Drug Administration
FGF: fibroblast growth factor
FGFR: fibroblast growth factor receptor
GBC: gallbladder cancer
GEMOX: gemcitabine plus oxaliplatin
HER: human epidermal growth factor receptor
HR: hazard ratio
i.v.: intravenously
iCCA: intrahepatic cholangiocarcinoma
ICI: immune checkpoints inhibitors
ICR: independent central review
IDH: isocitrate dehydrogenase
mAb: monoclonal antibody
MAPK: mitogen-activated protein kinase
MEK: mitogen-activated protein kinase/extracellular signal-regulated kinase kinase
MSI-H: microsatellite instability-high
NE: not estimable
NTRK: neurotrophic tyrosine receptor kinase
ORR: objective response rate
OS: overall survival
PD-1: programmed cell death protein 1
PD-L1: programmed cell death ligand 1
PFS: progression-free survival
PK: pharmacokinetics
PR: partial responses
q3w: every 3 weeks
QD: once a day
QoL: quality of life
RECIST: response evaluation criteria in solid tumors
SD: stable diseases
T-DM1: trastuzumab-emtansine
TRAEs: treatment-related adverse events
VEGF: vascular endothelial growth factor

## Declarations

### Copyright

© The Author(s) 2021.
